# Folate receptor-targeted mixed polysialic acid micelles for combating rheumatoid arthritis: *in vitro* and *in vivo* evaluation

**DOI:** 10.1080/10717544.2018.1472677

**Published:** 2018-05-23

**Authors:** Nan Zhang, Chunyu Xu, Na Li, Shasha Zhang, Lingling Fu, Xiao Chu, Haiying Hua, Xianghui Zeng, Yongxing Zhao

**Affiliations:** a Department of Pharmaceutics, School of Pharmaceutical Sciences, Zhengzhou University, Zhengzhou, HeNan, PR China;; b Key Laboratory of Targeting Therapy and Diagnosis for Critical Diseases, HeNan Province, Zhengzhou, HeNan, PR China;; c Key Laboratory of Advanced Pharmaceutical Technology, Ministry of Education of China, HeNan Province, Zhengzhou, HeNan, PR China;; d Institute of Medical and Pharmaceutical Sciences, Zhengzhou University, Zhengzhou, HeNan, PR China;; e Department of Pharmacy, University of Copenhagen, Copenhagen, Denmark

**Keywords:** Polysialic acid, micelles, rheumatoid arthritis, dexamethasone, folic acid

## Abstract

**Objective:** Rheumatoid arthritis (RA) is associated with chronic inflammation. The suppression of inflammation is key to the treatment of RA. Glucocorticoids (GCs) are classical anti-inflammatory drugs with several disadvantages such as poor water solubility and low specificity in the body. These disadvantages are the reasons for the quick elimination and side effects of GCs *in vivo*. Micelles are ideal carriers for GCs delivery to inflamed synovium. We set out to improve the targeting and pharmacokinetic profiles of GCs by preparing a targeting micelle system.

**Methods:** In this study, natural chlosterol (CC) and folic acid (FA) were used to fabricate polysialic acid (PSA) micelles for the targeted delivery of Dexamethasone (Dex). The biodistribution and therapeutic efficacy of the resulting micelles were evaluated *in vitro* and *in vivo*.

**Results:** PSA-CC and FA-PSA-CC micelles showed a size below 100 nm and a moderate negative charge. PSA-CC and FA-PSA-CC micelles could also enhance the intracellular uptake of Dex and the suppression of tumor necrosis factor-α (TNF-α) and interleukin-6 (IL-6) *in vitro* and *in vivo*. Arthritis mice showed reduced paw thickness and clinical arthritis index using PSA-CC and FA-PSA-CC micelle treatment. Micellized Dex demonstrated a 4 ∼ 5 fold longer elimination half-life and a 2 ∼ 3 folds higher bioavailability than commercial Dex injection. FA modification significantly improved the anti-inflammatory efficacy of PSA-CC micelles.

**Conclusion:** FA-PSA-CC micelles demonstrated significant advantages in terms of the suppression of inflammation and the treatment of inflammatory arthritis. These reliable and stable micelles possess a high potential to be transferred for clinical use.

## Introduction

Rheumatoid arthritis (RA) is one of the most severe autoimmune disordered diseases. RA has an incidence ∼1% world-wide and severe RA causes cartilage damage, bone resorption, organ injuries, and shortened life span. Despite modern medical progress, the pathogenesis of RA is unclear and the clinical diagnosis and treatment for RA stay in a semi-empirical phase. One of the most significant features of autoimmune diseases like RA is the activation of immune cells and immune responses, leading to chronic inflammation. In inflamed synovium, the co-stimulation of T cells and B cells activated macrophages to secrete various pro-inflammatory proteins such as TNF-α, IL-1, etc. The activated macrophages and T cells also stimulated synovial fibroblasts to express receptor activator for nuclear factor-κB ligand (RANKL) and matrix metalloproteinases (MMPs), which causes osteoclast activation, bone resorption, and cartilage degradation (Bader & Wagoner, 2010; Wagoner & Bader, 2012). Together, pro-inflammatory proteins and the activation of intracellular inflammatory signaling pathways result in the activation of multiple immune cells in inflamed synovium, which leads to chronic inflammation and irreversible tissue damage. The suppression of chronic inflammation is one of the most important strategies for RA treatment. Glucocorticoids (GCs) are cheap and efficient therapeutics for anti-inflammatory use in clinical setting over centuries. However, GCs like Dexamethasone (Dex) possess unspecific distribution and fast elimination after administration to the body. The lack of specificity to inflamed synovium leads to repeated administration in order to achieve an effective drug concentration, which usually causes various side effects to health tissues and reduces patient compliance. Improvements to the targeting and pharmacokinetic profiles of classical GCs are highly needed and significant in clinical setting.

Micelles are ideal carriers for poorly water-soluble molecules. Functional material have been used for the fabrication of micelles (Zhang et al., 2013) and various micelles have been developed in academic articles. However, only a few of these published micelles entered clinical studies so as to benefit patients. Past experience indicated that micelles with good safety, high efficacy, and easy synthesis and modification possess a higher possibility to be transferred into clinical use. PEGylation has been the most successful strategy to increase the solubility and prolong circulation time *in vivo*. However, PEG is hard to degrade, which may lead to the accumulation of high molecule weight PEG in the body. Some polysaccharides, like polysialic acid (PSA), (Drake et al., 2008) have similar properties to PEG and are biodegradable and excretable. Previously, PSA has been used for the development of micelles (Bader et al., 2011; Wilson et al., 2014; Zhang et al., 2014). As an alternative material to PEG, PSA is highly hydrophilic, biocompatible, and biodegradable. The FALT of PSA is similar to that of PEG, which indicates that the water absorbing ability of PSA is similar to that of PEG. PSA was firstly discovered on the surface of pathogenic bacteria. The thick coating of PSA on the cell wall facilitate bacteria to escape the host immune system and evade the host tissues. Further studies showed an anti-adhesive property of PSA (Elward & Gasque, 2003; Gregoriadis et al., 2005). PSA-conjugated insulin, asparaginase, and catalase have shown prolonged half-life *in vivo* (Fernandes & Gregoriadis, 1994, 1996, 1997, 2001; Jain et al., 2003; Gregoriadis et al., 2005). These reported properties show the potential of PSA as a stealth biomacromolecule for micelle development.

In this study, hydrophobic cholesteryl chloroformate (CC) was conjugated to PSA to prepare micelles for Dex loading. To enhance the targeting to inflamed synovium, folic acid (FA) modified PSA-CC micelles were also developed by forming a mixed micelle system. PSA-CC and FA-PSA-CC micelles were characterized in terms of their physiochemical properties and evaluated for their *in vitro* toxicity, anti-inflammatory efficacy, and interaction with immune cells. Furthermore, these micelles were monitored for their safety and examined for their *in vivo* distribution, the efficacy on the treatment of adjuvant induce arthritis (AIA) mice, and pharmacokinetics. Our study will demonstrate the potential of the developed micelles for RA treatment.

## Material and methods

### Material, cell culture and animals

PSA was purchased from HuBei HengLuYuan Technology Co., Ltd (HuBei, China). Sigma supplied cholesteryl chloroformate, tetrabutylammonium bromide, DOWEX 50WX2 ion-exchange resin, IR-780 iodide, and Dex. 1,2-Distearoyl-*sn*-glycero-3-phosphoethanolamine polyethylene glycol-folic acid (DSPE-PEG-FA) was obtained from Nanocs (New York, NY). Pyrene was purchased from Alfa Aesar (Ward Hill, MA). Lipopolysaccharide (LPS) and Complete Freund’s adjuvant were purchased from Sigma Corporation (Cram Ridge, NJ). HyClone Laboratories Inc. (South Logan, UT) supplied penicillin–streptomycin solution (100×). Gibco (Carlsbad, CA) provided fetal bovine serum (FBS). 0.25% trypsin, DMEM culture medium, DAPI solution were supplied by Solarbio (Beijing, China). ELISA kit was purchased from MultiSciences (LiankeBio) (Hangzhou, China). RAW 264.7 and Ges-1 cells were purchased from CoBioer (Nanjing, China).

### Synthesis of PSA-CC and FA-PSA-CC micelles

**Scheme 1.**
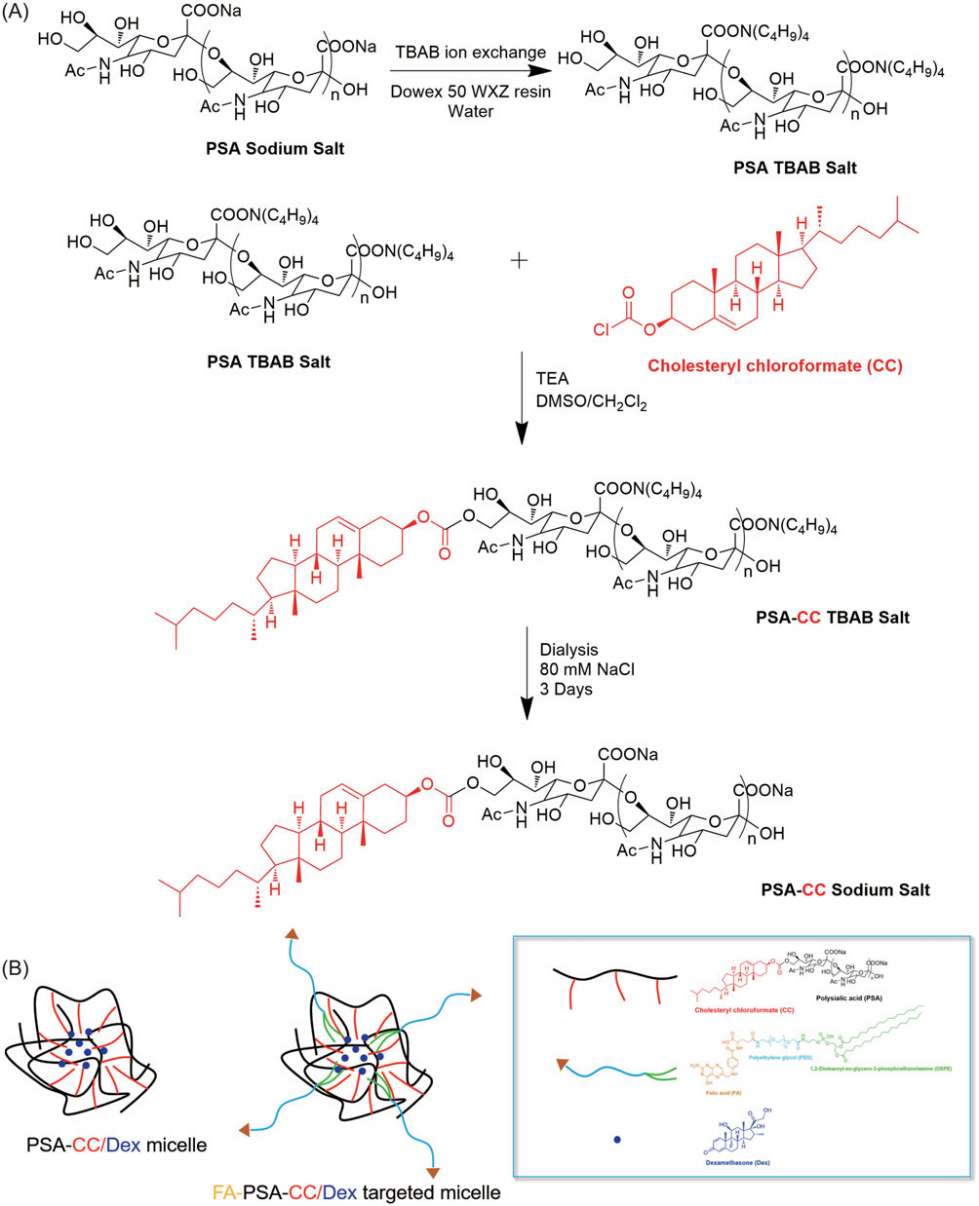
(A) Synthesis of PSA-CC micelles. (B) Illustration of the compositions of PSA-CC/Dex micelles and FA-PSA-CC/Dex micelles.

Synthesis of micelles were finished in DMSO/dichloromethane co-solvent system (Yao et al., 2014). The sodium ion of PSA was firstly exchanged to tetrabutylammonium to increase the solubility of PSA in DMSO according to a reported procedure (Wilson et al., 2014) (see details in Supplementary material). To synthesize PSA-CC, 16 mg of ion exchanged PSA (51.2 μmol sialic acid monomer) was added into 2.5 ml of anhydrous DMSO. PSA was dissolved well using sonication. About 10 μL triethylamine (TEA) was subsequently mixed into the DMSO solution. Simultaneously, 14 mg CC (0.031 mmol, 0.6 equiv. relative to sialic acid monomer) was dissolved in 1.5 ml of anhydrous dichloromethane. The dichloromethane solution was added slowly to the DMSO solution with stirring in N_2_ atmosphere. The mixture was stirred for 48 h at room temperature. Dichloromethane was removed by rotary evaporation. PSA-CC solution was subsequently filtered and dialyzed against 150 mM NaCl solution for 3 days to remove solvent residual and exchange tetrabutylammonium back to sodium. Purified PSA-CC was obtained by lyophilization and analyzed by FTIR and 1H-NMR. To prepare FA-PSA-CC, 2 ml dichloromethane that contained 1.5 mg DSPE-PEG-FA (MW5000) was added to the evaporated PSA-CC solution. About 2 ml DI water was subsequently added under vigorous stirring. The solution was rota-evaporated after 20 min stirring and was subsequently filtered and dialyzed against 150 mM NaCl solution for 3 days. Purified FA-PSA-CC was obtained by lyophilization and analyzed by FTIR and 1H-NMR (Scheme 1(A)).

### Characterization of PSA-CC and FA-PSA-CC micelles

#### Critical micelle concentration

Critical micelle concentration (CMC) was evaluated via steady-state ﬂuorescence using pyrene as a probe. Both PSA-CC and FA-PSA-CC solution were diluted into 11 samples (dilution from 1 mg/mL to 0.98 μg/mL). About 1 mL of each sample was mixed with 8.1 μL of a 1.24 × 10^−4^ M acetone solution of pyrene. The solutions were sonicated for 30 min at room temperature and heated at 65 °C under shaking for 4 h to achieve equilibration. After cooling in the dark overnight, excitation spectra were recorded at an emission wavelength of 390 nm using a RF-5301PC (Shimadzu Corporation, Kyoto, Japan) ﬂuorescence spectrophotometer.

#### Transmission electron microscopy

Lyophilized PSA-CC and FA-PSA-CC were dissolved in de-ionized water (DI) water to prepare the 0.1 mg/mL micelle solutions of PSA-CC and FA-PSA-CC. About 20 μL of the solutions was pipetted onto a carbon-coated copper grid (Beijing Xinxing Braim Technology Co., Ltd, Beijing, China). The excess micelle solution was removed, and the sample was observed under transmission electron microscopy (TEM; TecnaiG20; FEI, Hillsboro, OR).

#### Size and zeta potential

Size, polydispersity (PDI), and zeta potential of PSA-CC and FA-PSA-CC micelles were obtained using a Zetasizer Nano ZS (Malvern Instruments, Malvern, UK). Aqueous samples were prepared by dissolving 10 mg PSA-CC and FA-PSA-CC in 1 ml DI water and tested at 25 °C, respectively.

### Dex loading and release

One milligram of PSA-CC or FA-PSA-CC and 0.1–0.4 mg of Dex were added to acetonitrile and stirring for 1 h. The mixture was subsequently rota-evaporated at 65 °C to remove acetonitrile and formed a film at the bottom of flask. Two milliliter of DI water was refilled to the flask. The flask was sonicated for 5 min and filtered through 0.8 μm membrane. Loading capacity (LC) and loading efficiency (LE) were analyzed by high-performance liquid chromatography (HPLC) (Agilent 1100, Beijing, China) (column: Diamonsil C18, 5 μm, 200 × 4.6 mm; mobile phase: methanol/water 7/3; flow rate: 1 mL/min^−1^; UV detection wavelength: 240 nm; temperature: 25 °C; injected samples: 20 μL). About 250 μL of Dex-loaded micelles were mixed with 250 μL methanol and the samples were injected for HPLC analysis. Dex-loaded PSA-CC and FA-PSA-CC were also characterized for size, zeta potential, thermal stability and Dex release. Size and zeta potential were conducted as above. For thermal stability test, 1 mL of Dex-loaded micelles was incubated for 6 days at 37 °C with daily measurement of micelle size and PDI. Dex release was conducted in dialysis tubes. Briefly, 5 mL of Dex-loaded micelles was transferred into a dialysis tube (molecular weight cut off [MWCO] 12K–14K). The tube was submerged into 45 mL DI water (37 °C, pH 7.4). At set time points (10 min, 20 min, 40 min, 1 h, 2 h, 4 h, 6 h, 12 h, 24 h, 48 h and 72 h), 1 mL sink solution was taken as a sample and 1 mL fresh DI water was refilled. The samples were analyzed as above using HPLC.

### Cellular uptake of coumarin-loaded micelles by macrophages

To test intracellular delivery of molecules by micelles developed in this study, lipophilic coumarin that shows green fluorescence was loaded to PSA-CC and FA-PSA-CC micelles. Cellular internalization of coumarin was studied using A1 laser confocal microscopy (Nikon, Japan) on RAW 264.7 cells. Briefly, RAW 264.7 cells were seeded in 24 well plates with a cell density of 7.5 × 10^4^ cells per well. The cells were stimulated by LPS for 24 h. Coumarin solution, coumarin-loaded PSA-CC, and coumarin-loaded FA-PSA-CC were added to the wells and cultured for 0.5, 1, 2, and 4 h. Medium in each well was removed and cells were washed thrice with PBS after incubation. About 2 mL 4% paraformaldehyde was then added to each well. The cells were incubated for 10 min in the dark. Subsequently, the cells were washed with PBS thrice and 400 μL DAPI solution was used to stain cells for 5 min. The cells were washed thrice using PBS and observed under microscope.

To quantify the cellular uptake, coumarin, coumarin-loaded PSA-CC, and coumarin-loaded FA-PSA-CC were added to RAW 264.7 cells (a cell density of 3 × 10^5^ cells per well) and cultured for 4 h, respectively. Subsequently, cells were washed thrice using cold PBS. Five hundred microliter trypsin was added to each well and the detached cells were washed twice with PBS. Subsequently, the cells were centrifuged at 1000 r/min for 5 min. Cell pellet was washed and suspended with PBS for twice and analyzed using a BD FACS Canto II flow cytometer (BD Biosciences, Oxford, UK).

### In vitro anti-inflammatory efficacy in activated macrophages

RAW 264.7 cells in exponential phase of growth were seeded to 96-well plates at a cell density of 1.5 × 10^4^ cells per well. The cells were stimulated by 1 μg/ml LPS for 24 h to establish inflammatory cell models. Controls, Dex (0.1 mg/mL, 1 mg/mL), Dex (0.1 mg/mL) loaded PSA-CC, and Dex (0.1 mg/mL) loaded FA-PSA-CC were added to the inflammatory cells and incubated for 24 h. Cell supernatant was collected and tested by ELISA kit to determine the concentration of TNF-α and IL-6.

### In vivo anti-inflammatory efficacy in AIA mice

#### Biodistribution

IR-780 is a lipophilic and fluorescent molecule with good stability *in vivo*. IR-780 was loaded to PSA-CC and FA-PSA-CC micelles using the same method to Dex. AIA mice were randomly divided into three groups for the injection of IR-780 solution (0.2 mg IR-780 per mg mice, 100 μL), IR-780 (0.2 mg IR-780 per mg mice, 100 μL) loaded PSA-CC, and IR-780 (0.2 mg IR-780 per mg mice, 100 μL) loaded FA-PSA-CC. At 2, 6, 16, and 24 h after tail veil injection, the mice were anesthetized and observed for dynamic fluorescent distribution in mice using In Vivo Imaging System FX PRO (excitation wavelength 770 nm, emission wavelength 840 nm, X-ray exposure time 30 s) (Bruker; Billerica, MA).

#### Efficacy study

SPF Swiss male mice (body weight around 23–27 g) were purchased from the Experimental Animal Center of Zhengzhou University. Animal care and experiments were performed with the approval of the animal ethical committee of Zhengzhou University (Zhengzhou, China), according to the requirements of the National Act on the Use of Experimental Animals (China). All animals were kept in a favorable environment and were acclimated at 25 °C and 55% of humidity under natural light/dark conditions, with free access to a rodent diet and water. The experimental animals were acclimated for 1 week before the beginning of the study. Experimental mice were injected with 40 μL Freund’s adjuvant (10 mg/mL). Mice with inflamed paw were selected after 15 days and randomly divided into five groups for the injection of 200 μL saline every other day to normal mice, 200 μL saline every other day to inflamed mice, Dex solution (0.02 mg Dex per mouse every other day), Dex-loaded PSA-CC solution (0.02 mg Dex per mouse every other day), and Dex-loaded FA-PSA-CC solution (0.02 mg Dex per mouse every other day). The treatment lasted for 10 days. Paw thickness, paw inflammation, and paw flexibility were recorded every other day. Arthritis clinical index of mouse joints was calculated based on the paw thickness, paw inflammation, and paw flexibility. The mice were sacrificed after treatment (at Day 10) and 0.5 mL of blood samples were collected. ELISA kit was used to determine the concentration of TNF-α and IL-6 in blood. The joints of mice were collected and sliced for histological examination. The histological slices were dyed with HE and examined by a Leica DM3000 light microscope (Leica Microsystems, CMS GmbH, Wetzlar, Germany).

### Pharmacokinetics study in AIA rats

The pharmacokinetic (PK) study was conducted on Sprague-Dawley rats. The rats weighting 200 ± 20 g were injected with 100 μL Freund’s adjuvant (10 mg/mL) subcutaneously at the base of rat tail. Rats with inflamed paw were selected after 15 days and were divided into three groups for the injection of Dex solution (0.05 mg Dex in 0.5 mL per rat), Dex-loaded PSA-CC solution (0.05 mg Dex in 0.5 mL per rat), and Dex-loaded FA-PSA-CC solution (0.05 mg Dex in 0.5 mL per rat). Blood samples (0.3 mL) were collected into heparinized Eppendorf tubes at 0.083, 0.25, 0.5, 1, 2, 4, 8, 12, 24, and 48 h after tail vein administration. Blood plasma was obtained by centrifuging at 4000 rpm for 10 min. Subsequently, the plasma (0.1 mL) was mixed with 0.1 mL methanol, shaken by a vortex mixer for 3 min, and centrifuged at 3500 rpm for 15 min. The supernatant was determined by HPLC (as described above) for Dex concentration. Pharmacokinetics parameters were calculated by PK-Solver software (Redmond, WA).

### Safety of micelles in AIA mice

On Day 24 after induction, the levels of aspartate aminotransferase (AST) and alanine transaminase (ALT) in blood were determined in AIA mice treated by Dex solution, Dex-loaded PSA-CC, and Dex-loaded FA-PSA-CC. These levels were measured using an automatic biochemical analyzer. At the same time, white blood cell counts and lymphocyte counts were determined using a standard hematology method.

## Results

### Synthesis and characterization of PSA-CC and FA-PSA-CC micelles

**Figure 1.**
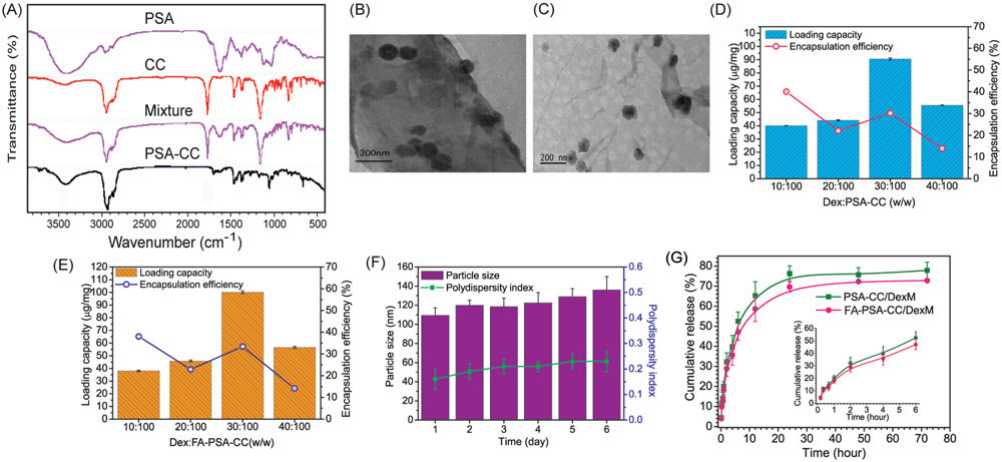
Characterization of the micelles. (A) FTIR spectrum of micelles and compositions for the blank micelles. (B) TEM image of PSA-CC micelles. (C) TEM image of FA-PSA-CC micelles. (D) Loading capacity and loading efficiency of PSA-CC/Dex micelles. (E) Loading capacity and loading efficiency of FA-PSA-CC/Dex micelles. (F) Particle size and PDI of FA-PSA-CC/Dex micelles. (G) Dex release from PSA-CC micelles and FA-PSA-CC micelles.

FTIR spectrums indicated hydroxyl and amide functional group and of PSA around 3400 and 1650 cm^−1^, respectively. CC showed featured acyl chloride peak at 1790 cm^−1^, carbon–hydrogen bond at 2950 cm^−1^, and double peaks for C(CH_3_)_2_ at 1400 cm^−1^. Successful conjugation of CC to PSA destroyed acyl chloride group and resulted in the peak disappearance at 1790 cm^−1^. (Figure 1(A)) NMR result of PSA-CC also confirmed the appearance of hydrogens that belonged to CC at 0.5–1.5 ppm. (Supplementary Figure S1) CMC results indicated that PSA-CC and FA-PSA-CC started to form micelles at the concentration of 46.2 ± 3.9 and 32.1 ± 5.2 μg/mL, respectively (Supplementary Figure S2(A,B)). TEM showed a round shape of PSA-CC and FA-PSA-CC micelles with the size 80–100 nm (Figure 1(B,C)). TEM results were consistent with dynamic light scattering results. The size of PSA-CC and FA-PSA-CC micelles was 93.8 ± 13.4 and 83.8 ± 13.4 nm respectively using zeta-sizer. Both of PSA-CC and FA-PSA-CC micelles showed a sufficient negative charge and small PDI, indicating a high stability in solution (Supplementary Figure S2(C)). With Dex loading, the size of PSA-CC and FA-PSA-CC micelles increased slightly (Supplementary Figure S3).

### Dex loading and release, and micelle stability

LC and LE of Dex to micelles were optimized by loading varied amount of Dex. Results showed that a highest LC of Dex to PSA-CC and FA-PSA-CC micelles was obtained when the ratio of Dex to micelles is 0.3–1.0 mg. At this ratio, ∼0.1 mg of Dex was encapsulated by 1 mg micelle and ∼33.3% of initially added Dex was loaded into micelles (Figure 1(D,E)). Slight size and PDI increase of FA-PSA-CC/Dex micelles was observed during the 6-day incubation at 37 °C (Figure 1(F)). Figure 1(G) showed the release profile of Dex from PSA-CC and FA-PSA-CC micelles. Around half of loaded Dex was released at 6 h and 70–80% loaded Dex was released at 72 h.

### In vitro study

**Figure 2.**
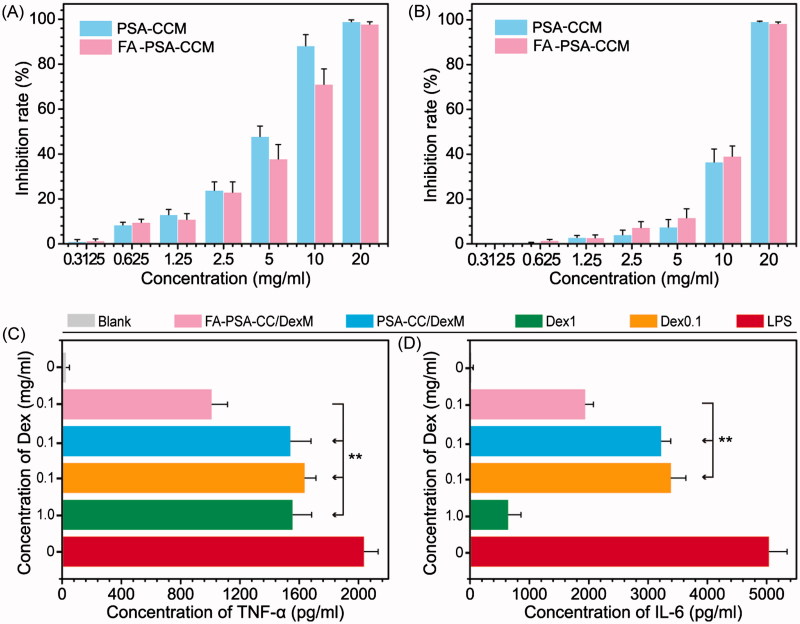
Inhibition rate of PSA-CC micelles and FA-PSA-CC micelles on RAW 264.7 cells (A) and GES-1 cells. (B) Concentration of TNF-α (C) and IL-6 (D) in RAW 264.7 cells with listed treatment.

**Figure 3.**
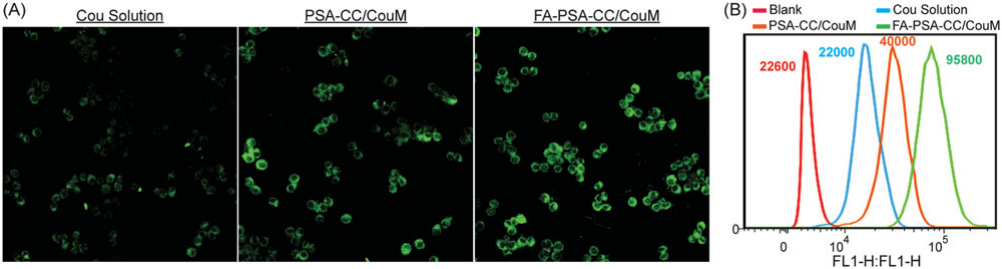
Cellular uptake of PSA-CC micelles and FA-PSA-CC micelles. (A) Fluorescent images of coumarin and coumarin-loaded micelle treated macrophages. (B) Flow cytometric graphs of fluorescent intensity of coumarin in macrophages that treated as listed.

The cytotoxicity of PSA-CC and FA-PSA-CC micelles was tested in RAW 264.7 and GES-1 cells. IC_50_ values of PSA-CC micelles to RAW 264.7 and GES-1 cells were 4.98 ± 0.73 and 9.97 ± 0.98 mg/mL, respectively; IC_50_ values of FA-PSA-CC micelles to RAW 264.7 and GES-1 cells were 5.85 ± 0.58 and 11.69 ± 0.48 mg/mL, respectively. Micelles (1 mg/mL) showed insignificant cytotoxicity to RAW 264.7 and GES-1 cells and thus were considered to be safe for *in vitro* study (Figure 2(A,B)). Dex, Dex-loaded PSA-CC and FA-PSA-CC micelles were assayed for their anti-inflammatory responses. PSA-CC and FA-PSA-CC did not induce the expression of TNF-α and IL-6 (Supplementary Figure S4). All treatment could reduce the production of TNF-α and IL-6. Dex-loaded FA-PSA-CC (0.1 mg/mL of Dex) resulted in a higher reduction of TNF-α and IL-6 than Dex (0.1 mg/mL) and Dex-loaded PSA-CC (0.1 mg/mL of Dex). FA-PSA-CC could increase the anti-inflammatory efficacy of Dex (Figure 2(C,D)). FA-PSA-CC group showed the strongest fluorescence in cells, indicating that FA-PSA-CC micelles possessed the most intracellular delivery of coumarin (Supplementary Figure S4) Further internalization studies showed the most coumarin accumulation in cytoplasm (Supplementary Figure S5). Analysis using flow cytometer confirmed that the ratio of coumarin delivered by PSA-CC to free coumarin is 1.81, and the ratio of coumarin delivered by FA-PSA-CC to free coumarin is 4.35 (Figure 3).

### In vivo study

**Figure 4.**
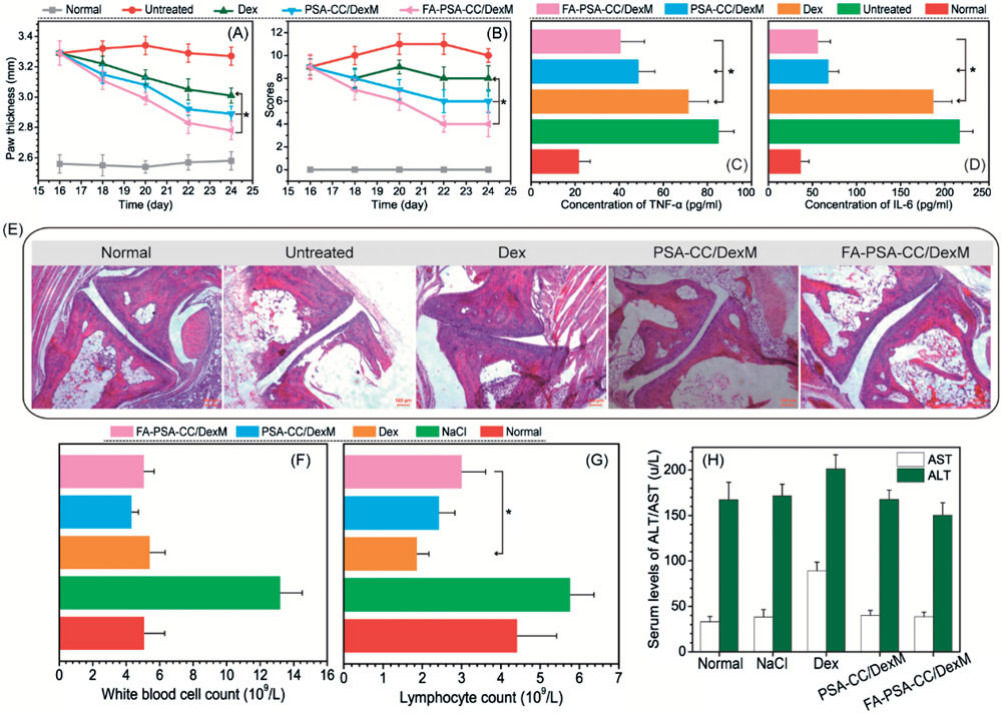
Paw thickness (A) and clinical index (B) of mice with listed treatment. Concentration of serum TNF-α (C) and IL-6 (D) in AIA mice with listed treatment. (E) Images of histological slides from AIA mice with listed treatment. Safety evaluation: the concentration of (F) White blood cell, (G) lymphocyte, (H) AST, and ALT in blood with listed treatment.

Inflammatory mice were treated every other day for 10 days. Paw thickness and other inflammatory parameters were measured every other day. Clinical arthritis scores were calculated based on the sum of paw thickness, paw inflammation, and paw flexibility. Figure 4(A,B) showed that Dex and Dex-loaded PSA-CC and FA-PSA-CC micelles caused a significant decrease of paw thickness and clinical arthritis scores. Dex-loaded FA-PSA-CC micelles showed the highest reduction of paw thickness and clinical arthritis scores. Blood samples were obtained and tested after the 10 days’ treatment. The serum concentration of TNF-α and IL-6 in mice that were treated by Dex-loaded PSA-CC and FA-PSA-CC micelles was significantly reduced compared to mice treated by Dex solution (Figure 4(C,D)). Pathological slides showed that normal mice and mice treated with Dex-loaded PSA-CC and FA-PSA-CC micelles possessed smooth cartilages and no pannus invasion, whereas untreated mice showed cartilage and bone damage. Mice treated by Dex demonstrated an abnormal structure of joint (Figure 4(E)).

The number of white blood cells in mice treated with Dex, PSA-CC/DexM and FA-PSA-CC/DexM is similar to that in normal mice. The number of lymphocytes in mice treated with Dex, PSA-CC/DexM and FA-PSA-CC/DexM is less than that in normal mice. However, FA-PSA-CC/DexM reduced the decrease of lymphocytes caused by Dex and the number of lymphocytes in FA-PSA-CC/DexM treated mice was closer to that in normal mice (Figure 4(F,G)). AST and ALT are indicative parameters for liver function. Mice treated by Dex-loaded micelles showed similar level of AST and ALT to that in normal mice, whereas free Dex treatment caused an increase of AST and ALT in plasma. The results indicated that a high biocompatibility of the developed micelles (Figure 4(H)).

**Figure 5.**
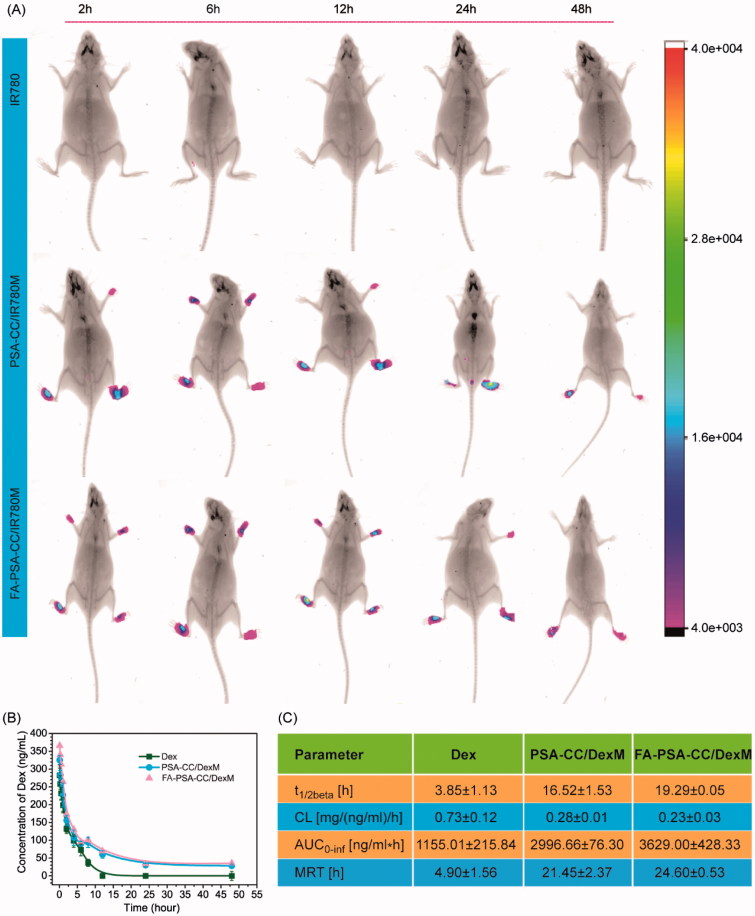
(A) Biodistribution of IR780, PSA-CC/IR780 micelles, and FA-PSA-CC/IR780 micelles in AIA mice. (B) Concentration of serum Dex in AIA mice with listed treatment. (C) Key pharmacokinetics parameters of Dex, PSA-CC/Dex micelles, and FA-PSA-CC/Dex micelles in AIA mice.

The biodistribution of PSA-CC and FA-PSA-CC micelles was studied in AIA mice. The results showed that fluorescence was not observed in mice injected with free IR-780, whereas strong fluorescence was shown in the paw of mice that were injected with IR780-loaded PSA-CC micelles (PSA-CC/IR780M) and FA-PSA-CC micelles (FA-PSA-CC/IR780M). Mice treated with FA-PSA-CC/IR780M generally demonstrated stronger fluorescence than that treated by PSA-CC/IR780M (Figure 5(A)). The pharmacokinetics (PK) of Dex-loaded micelles was also explored. After injection, blood samples were collected at set time points for the study of PK in AIA rats. At 12 h, Dex was barely detected in the blood of rats that were injected with free Dex, whereas the blood concentration of Dex was still detectable at 48 h in rats that were injected with Dex-loaded micelles. PK parameters were calculated using the blood concentration of Dex. Results showed that PSA-CC and FA-PSA-CC micelles have significantly increased the *t*_1/2β,_ *AUC*, and MRT of Dex in AIA rats (Figure 5(B,C)).

## Discussion

Many effective anti-rheumatic drugs are small hydrophobic molecules, such as methotrexate, Dex, and cyclosporine A. However, their use is gradually replaced by new and expensive therapies such as TNF-α inhibitors and IL-1 inhibitors because the poor *in vivo* PK/PD and high risk of side effects of the traditional drugs. In this study, we aim to improve the use of traditional drugs via drug delivery technology. Quan et al. (2014) have previously conducted a comparison study of Dex-containing polymers, micelles, and liposomes for inflammatory arthritis treatment (Quan et al., 2014). The results showed that Dex-loaded micelles are one of the best treatments in protecting the bone and reducing joint swelling, disease activity, and histology score of the joints. Compared to liposomes, micelles with low CMC possess reduced pre-leakage of small hydrophobic drug molecules. Micelles are more advantageous than dendrimers and polymeric conjugates because the loading of GC to micelles does not need chemical reaction. For small hydrophobic drug molecules, micelles provide a higher drug loading capacity and better protection than polymeric nanoparticles. Therefore, micelles are chosen in this study to improve Dex delivery. Compared to other reported micelles, our micelles use PSA to replace PEG for prolonged circulation *in vivo*, and the amphiphilic FA is used to increase the stability, loading capacity, and active targeting of our mixed micelles. Such self-assembled mixed micelles are more easily to be prepared than other advanced micelles that need further synthesis. What’s more important, this is the first study to demonstrate the advantages of PSA-based micelles for Dex delivery *in vitro* and *in vivo*.

PEG has been the gold standard to increase the *in vivo* circulation of therapeutics. However, PEG is undegradable *in vivo*. Long term use of PEG may cause toxicity and immune responses (Semple et al., 2005; Webster et al., 2007; Hamad et al., 2008). Biodegradable macromolecules that have similar properties to PEG are potential alternatives to PEG for the next generation of nanomedicines (Bader & Wardwell, 2014). Inspired by the natural stealth property of PSA, PSA-based drug delivery systems have been developed to reduce the *in vivo* clearance and prolong the *in vivo* circulation of drugs. Gregoriadis (2005) is a pioneer for the studies of sialylated and polysialylated therapeutics (Fernandes & Gregoriadis, 1994, 1996, 1997, 2001; Jain et al., 2003) and have founded Xenetic Biosciences, Inc. (Lexington, MA) in 1997 based on these technologies (PolyXen^TM^). PolyXen^TM^ has been a clinically proved platform technology to improve the circulation and other pharmacological properties of protein or peptide therapeutics. Although sialylated and polysialylated therapeutics demonstrated better efficacy, sialic acid or PSA-drug conjugates are limited by their small size (quick clearance) and low drug loading compared to micelles, nanoparticles, and liposomes. Our group have previously developed PSA-based micelles for the treatment of rheumatoid arthritis (Bader et al., 2011). PSA-decylamine (DA) micelle failed because of its high cytotoxicity. PSA-polycaprolactone (PCL) micelles are biocompatible but need multi-step chemical synthesis. A biocompatible micelle system that can be synthesized via one step and controlled precisely for its compositions is highly needed.

Cholesterol (CC), a natural and essential molecule for the body, was selected as the hydrophobic moiety of our micelles. CC was conjugated to PSA via one-step reaction. Amphiphilic DSPE-PEG-FA was mixed with PSA-CC to develop the targeted FA-PSA-CC micelles (Scheme 1(B)). PSA-CC and FA-PSA-CC formed micelles at a lower concentration than PSA-DA micelles and PSA-PCL micelles, indicating that PSA-CC and FA-PSA-CC micelles possessed a better physiochemical stability. PSA-CC and FA-PSA-CC micelles also demonstrated good biocompatibility *in vitro* and *in vivo*. A longer accumulation of PSA-CC-dye and FA-PSA-CC-dye micelles was observed in AIA mice (Figure 5(A)). PSA-CC and FA-PSA-CC micelles also significantly reduced Dex clearance from AIA rats and increased *AUC* of Dex in AIA rats (Figure 5(B,C)). For the first time, PSA-based micelles were shown to improve the pharmacokinetics of drugs *in vivo*.

In the period of inflammation, activated macrophages show high expression of folate receptor-β (FRβ) while quiescent resident macrophages have low or no expression of folate receptor. FA possesses high affinity to FRβ and has been used as an efficient ligand for targeting to activated macrophages (Rollett et al., 2012; Yang et al., 2014). Besides, FA itself has been shown to inhibit LPS-induced inflammatory response in RAW 264.7 macrophages by suppressing MAPKs and NF-κB activation (Feng et al., 2011). Therefore, the addition of FA to the micelles would facilitate FRβ-mediated cellular uptake by macrophages and FA induced suppression of inflammation in macrophages.

Physical mixing is an easy method to prepared FA-modified micelles. Mixing method avoid the introduction of additional toxic chemicals from conjugation reaction and the process of chemical residual removal. Instead of chemical conjugation of FA to PSA-CC, mixing of DSPE-PEG-FA with PSA-CC formed a targeted micelle with smaller size, lower CMC, and slower release of Dex, indicating that FA-PSA-CC micelles have a higher stability and tighter structure than PSA-CC micelles. FA was exposed to the surface of the mixed micelles. FA-PSA-CC micelles showed more intracellular delivery of therapeutics and could enhance the suppression of pro-inflammatory proteins *in vitro* and *in vivo*. The evaluation of paw thickness, clinical arthritis scores, and joint structure showed a better anti-inflammatory arthritis efficacy of FA-PSA-CC micelles than PSA-CC micelles. Therefore, the FA-PSA-CC mixed micelles possessed an easy preparation as well as high anti-inflammatory efficacy.

High drug loading is essential to successful drug delivery. Previous studies reported a Dex loading around 2% to PEG-PCL micelles and PEG-PCL/PEG-PEI hybrid micelles (Wang et al., 2016, 2017). The efficacy of Dex is dose-dependent. Therefore, a higher loading of Dex to micelles can reduce the amount of injected micelles into the body. By using an improved Dex loading method, loading capacity of Dex was increased to 10% in this study. The developed FA-PSA-CC/Dex micelles also demonstrated a longer elimination half-life and a higher AUC than previously reported Dex-loaded micelles (Wang et al., 2016), indicating that FA-PSA-CC/Dex micelles have more potential to be successfully transferred into clinical use.

## Conclusion

Compared to the discovery of new drugs, micellization of drugs on market is an easier and more cost-effective strategy to improve the efficacy and safety of treatments. This study micellized a classical anti-inflammatory drug-Dex with the biocompatible PSA and CC for the treatment of rheumatoid arthritis. Micellized Dex showed a proper size for passive targeting to inflamed joints and high targeting to macrophages that highly expressed FA receptor. Micellized Dex also demonstrated good stability, effective suppression of key pro-inflammatory proteins, improved PK of Dex, and good safety *in vitro* and *in vivo*. These results indicate that the micellized Dex is a potential strategy to improve the treatment of rheumatoid arthritis in clinical setting.

## Supplementary Material

Supplemental Material

## References

[CIT0001] Bader RA, Silvers AL, Zhang N. (2011). Polysialic acid-based micelles for encapsulation of hydrophobic drugs. Biomacromolecules 12:314–20. 21218771 10.1021/bm1008603

[CIT0002] Bader RA, Wagoner KL. (2010). Modulation of the response of rheumatoid arthritis synovial fibroblasts to proinflammatory stimulants with cyclic tensile strain. Cytokine 51:35–41. 20399680 10.1016/j.cyto.2010.03.015

[CIT0003] Bader RA, Wardwell PR. (2014). Polysialic acid: overcoming the hurdles of drug delivery. Ther Deliv 5:235–7. 24592947 10.4155/tde.13.153

[CIT0004] Drake PM, Nathan JK, Stock CM, et al (2008). Polysialic acid, a glycan with highly restricted expression, is found on human and murine leukocytes and modulates immune responses. J Immunol 181:6850–8. 18981104 10.4049/jimmunol.181.10.6850 PMC2718713

[CIT0005] Elward K, Gasque P. (2003). “Eat me” and “don’t eat me” signals govern the innate immune response and tissue repair in the CNS: emphasis on the critical role of the complement system. Mol Immunol 40:85–94. 12914815 10.1016/s0161-5890(03)00109-3

[CIT0006] Feng D, Zhou Y, Xia M, Ma J. (2011). Folic acid inhibits lipopolysaccharide-induced inflammatory response in RAW264.7 macrophages by suppressing MAPKs and NF-kappa B activation. Inflamm Res 60:817–22. 21528358 10.1007/s00011-011-0337-2

[CIT0007] Fernandes AI, Gregoriadis G. (1994). FC41 catalase-polysialic acid conjugates. Eur J Pharm Sci 2:111.

[CIT0008] Fernandes AI, Gregoriadis G. (1996). Synthesis, characterization and properties of sialylated catalase. Biochim Biophys Acta 1293:90–6. 8652633 10.1016/0167-4838(95)00227-8

[CIT0009] Fernandes AI, Gregoriadis G. (1997). Polysialylated asparaginase: preparation, activity and pharmacokinetics. Biochim Biophys Acta 1341:26–34. 9300806 10.1016/s0167-4838(97)00056-3

[CIT0010] Fernandes AI, Gregoriadis G. (2001). The effect of polysialylation on the immunogenicity and antigenicity of asparaginase: implication in its pharmacokinetics. Int J Pharm 217:215–24. 11292557 10.1016/s0378-5173(01)00603-2

[CIT0011] Gregoriadis G, Jain S, Papaioannou I, Laing P. (2005). Improving the therapeutic efficacy of peptides and proteins: a role for polysialic acids. Int J Pharm 300:125–30. 16046256 10.1016/j.ijpharm.2005.06.007

[CIT0012] Hamad I, Hunter AC, Szebeni J, Moghimi SM. (2008). Poly(ethylene glycol)s generate complement activation products in human serum through increased alternative pathway turnover and a MASP-2-dependent process. Mol Immunol 46:225–32. 18849076 10.1016/j.molimm.2008.08.276

[CIT0013] Jain S, Hreczuk-Hirst DH, Mccormack B, et al (2003). Polysialylated insulin: synthesis, characterization and biological activity *in vivo* . Biochim Biophys Acta 1622:42–9. 12829260 10.1016/s0304-4165(03)00116-8

[CIT0014] Quan L, Zhang Y, Crielaard BJ, et al (2014). Nanomedicines for inflammatory arthritis: head-to-head comparison of glucocorticoid-containing polymers, micelles, and liposomes. ACS Nano 8:458–66. 24341611 10.1021/nn4048205 PMC3947749

[CIT0015] Rollett A, Reiter T, Nogueira P, et al (2012). Folic acid-functionalized human serum albumin nanocapsules for targeted drug delivery to chronically activated macrophages. Int J Pharm 427:460–6. 22374516 10.1016/j.ijpharm.2012.02.028

[CIT0016] Semple SC, Harasym TO, Clow KA, et al (2005). Immunogenicity and rapid blood clearance of liposomes containing polyethylene glycol-lipid conjugates and nucleic Acid. J Pharmacol Exp Ther 312:1020–6. 15525796 10.1124/jpet.104.078113

[CIT0017] Wagoner KL, Bader RA. (2012). Evaluation of SV40-transformed synovial fibroblasts in the study of rheumatoid arthritis pathogenesis. Rheumatol Int 32:1885–91. 21445545 10.1007/s00296-011-1913-z

[CIT0018] Wang Q, Jiang J, Chen W, et al (2016). Targeted delivery of low-dose dexamethasone using PCL-PEG micelles for effective treatment of rheumatoid arthritis. J Control Release 230:64–72. 27057749 10.1016/j.jconrel.2016.03.035

[CIT0019] Wang Q, Jiang H, Li Y, et al (2017). Targeting NF-kB signaling with polymeric hybrid micelles that co-deliver siRNA and dexamethasone for arthritis therapy. Biomaterials 122:10–22. 28107661 10.1016/j.biomaterials.2017.01.008

[CIT0020] Webster R, Didier E, Harris P, et al (2007). PEGylated proteins: evaluation of their safety in the absence of definitive metabolism studies. Drug Metab Dispos 35:9–16. 17020954 10.1124/dmd.106.012419

[CIT0021] Wilson DR, Zhang N, Silvers AL, et al (2014). Synthesis and evaluation of cyclosporine A-loaded polysialic acid-polycaprolactone micelles for rheumatoid arthritis. Eur J Pharm Sci 51:146–56. 24075961 10.1016/j.ejps.2013.09.013

[CIT0022] Yang C, Gao S, Kjems J. (2014). Folic acid conjugated chitosan for targeted delivery of siRNA to activated macrophages *in vitro* and *in vivo* . J Mater Chem B 2:8608–15. 10.1039/c4tb01374c 32262219

[CIT0023] Yao X, Chen L, Chen X, et al (2014). Intercellular pH-responsive histidine modified dextran-g-cholesterol micelle for anticancer drug delivery. Colloids Surf B Biointerfaces 121:36–43. 24929531 10.1016/j.colsurfb.2014.05.032

[CIT0024] Zhang N, Wardwell PR, Bader RA. (2013). Polysaccharide-based micelles for drug delivery. Pharmaceutics 5:329–52. 24300453 10.3390/pharmaceutics5020329 PMC3834947

[CIT0025] Zhang N, Wardwell PR, Bader RA. (2014). *In vitro* efficacy of polysaccharide-based nanoparticles containing disease-modifying antirheumatic drugs. Pharm Res 31:2326–34. 24595497 10.1007/s11095-014-1329-z

