# Supplementary material for: Folate receptor-targeted mixed polysialic acid micelles for combating rheumatoid arthritis: in vitro and in vivo evaluation
Source: Drug Deliv. 2018 May 23;25(1):1182–91. doi: 10.1080/10717544.2018.1472677 (PMC6060703; doi:10.1080/10717544.2018.1472677)
Supplement: Supplemental Material [file IDRD_A_1472677_SM5423.docx]

**Folate receptor targeted mixed polysialic acid micelles for combating rheumatoid arthritis: *in vitro* and *in vivo* evaluation**

Nan Zhang, Chunyu Xu, Na Li, Shasha Zhang, Lingling Fu, Xiao Chu, Haiying Hua, Xianghui Zeng, Yongxing Zhao

**Synthesis of PSA-TBA**

600 mg Dowex 50 WXZ and 750 mg tetrabutylammonium bromide (2.26 mmol) (TBA) were mixed in 15 ml of DI water, and the mixture was stirred gently for 1 h. The Dowex 50 WXZ was washed several times with DI water to remove unbounded TBA. The washed Dowex was transferred into 10 ml of 2% aqueous (w/w) PSA (200 mg, 0.65 mmol sialic acid monomer), and the solution was stirred for 2 h at room temperature. The resin was separated from the liquid by centrifugation at 1000 rpm for 5 min. PSA solution was collected and ion exchanged PSA was isolated via lyophilization.

**Cytotoxicity of micelles to macrophages and human gastric epithelium cells**

RAW 264.7 (1.5×10^4^ cells per well) and GES-1 (1.5×10^4^ cells per well) cells were in exponential phase of growth and were cultured for 24 h at 37 °C in 96 well plates. PSA-Chol and FA-PSA-Chol were dissolved in culture medium and the solutions were filtered through 0.22 μm membrane before the addition to 96 well plate. (concentration of PSA-Chol and FA-PSA-Chol: 0.3125 mg/mL~20 mg/mL) The cells were incubated with micelles for 24 h. Subsequently, culture medium was removed and 200 μL cell culture medium with CCK8 (2.5%, v/v) was added to each well. After 4 h incubated, plates were read for OD value at 450 nm by plate reader. Cell inhibition was calculated as:

Cell inhibition (%) = [1 - $\frac{\left( {OD}_{Control}-{OD}_{Blank} \right)-({OD}_{Treatment}-{OD}_{Blank})}{({OD}_{Control}-{OD}_{Blank})}$
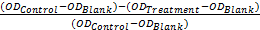
] × 100


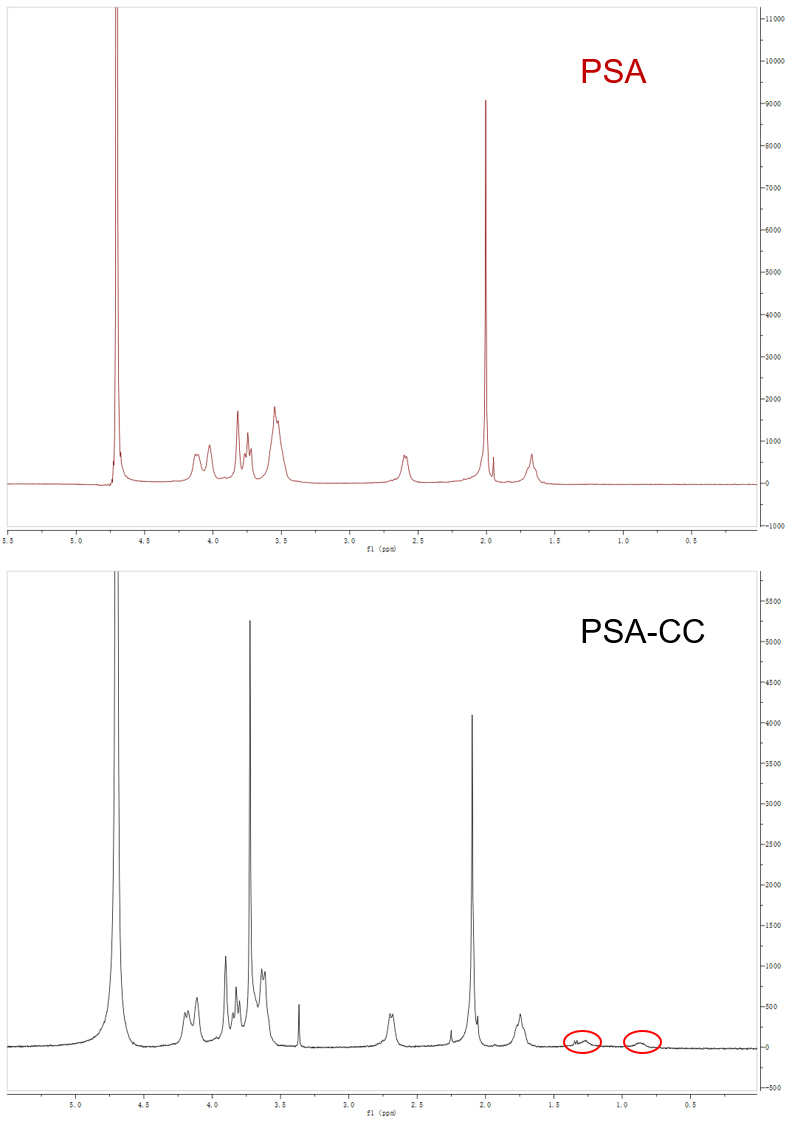


Figure S1. ^1^H-NMR spectrum of PSA and PSA-CC. Red circles represented hydrogen belonged to CC.


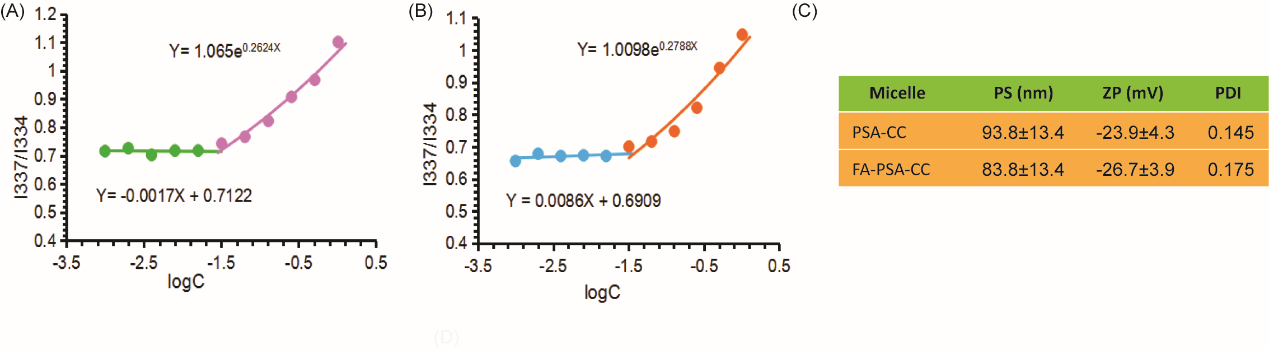


Figure S2. (A) Critical micelle concentration plot of PSA-CC (B) Critical micelle concentration plot of FA-PSA-CC (C) Size, zeta potential, and PDI of PSA-CC micelles and FA-PSA-CC micelles


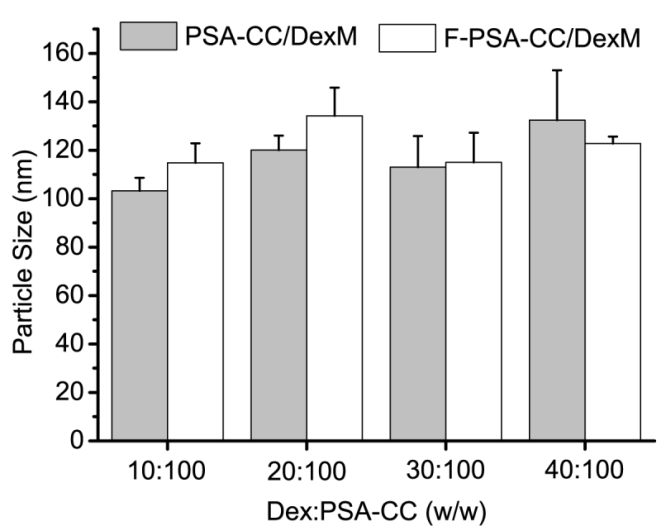


Figure S3. Determination of Particle Size of Drug-loaded Micelles by Self-assembled solvent volatilization


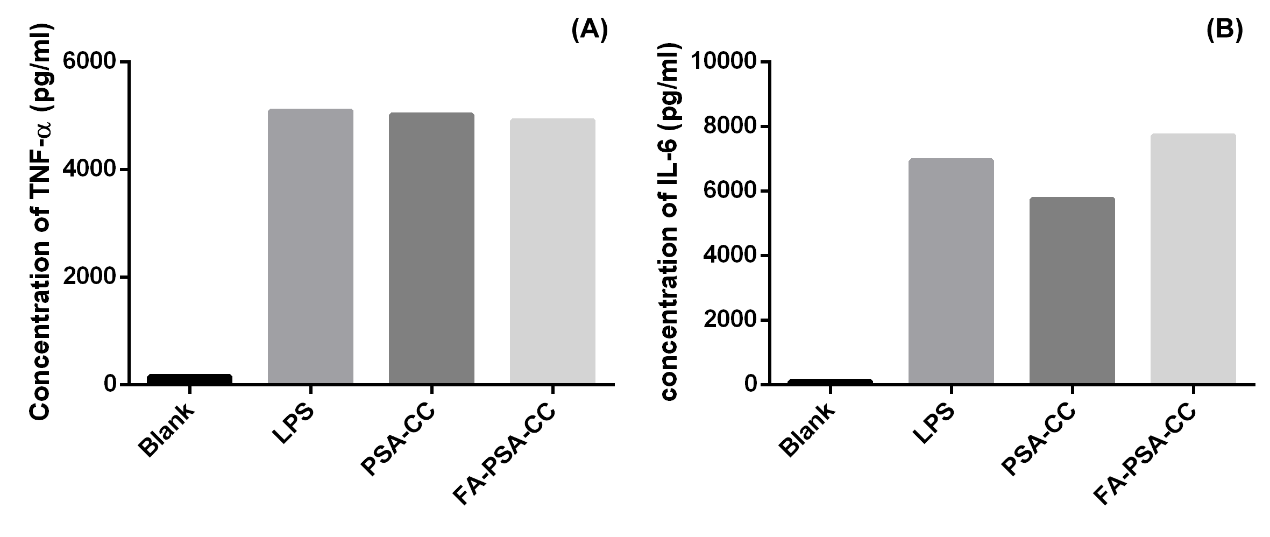


Figure S4. Concentration of TNF-α (A) and IL-6 (B) in RAW 264.7 cells with listed treatment.


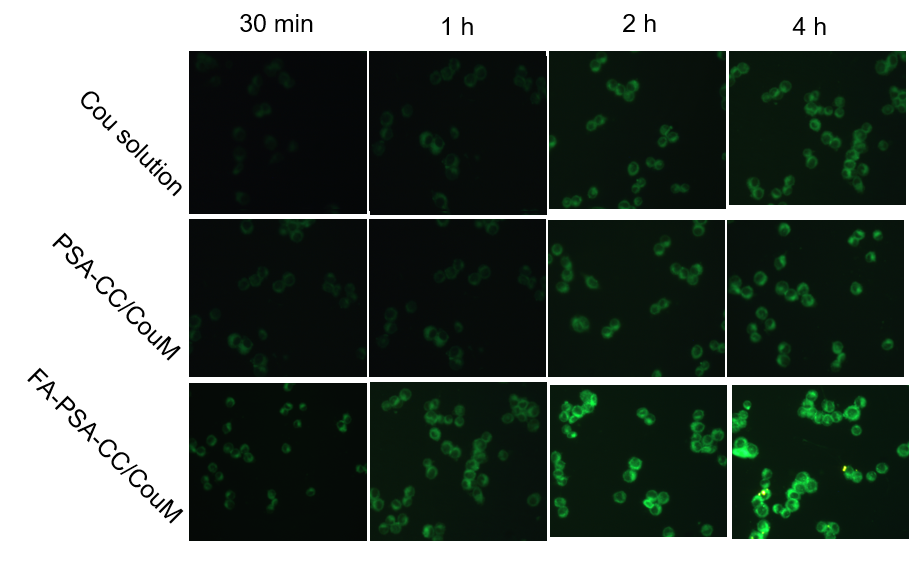


Figure S5. Fluorescent images of RAW 264.7 cells that indicated the internalization of listed formulation with incubation from 30 min to 4 h.


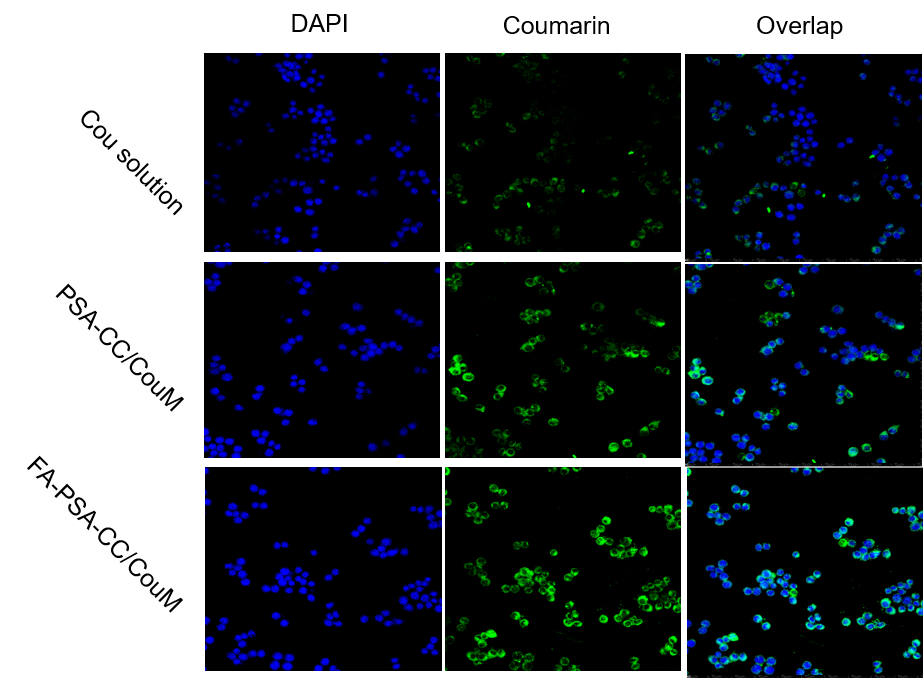


Figure S6. Fluorescent images of RAW 264.7 cells that indicated the location of coumarin and micelles after 4 h incubation.
